# Attitudes towards the Utilization of Intraosseous Access in Prehospital and Emergency Medicine Nursing Personnel

**DOI:** 10.3390/medicina58081086

**Published:** 2022-08-12

**Authors:** Matjaž Žunkovič, Andrej Markota, Amadeus Lešnik

**Affiliations:** 1 Community Health Care Centre Slovenska Bistrica, Partizanska ulica 30, 2310 Slovenska Bistrica, Slovenia; 2 Medical Intensive Care Unit, University Medical Centre Maribor, Ljubljanska ulica 5, 2000 Maribor, Slovenia; 3 Medical Emergency Department, University Medical Centre Maribor, Ljubljanska ulica 5, 2000 Maribor, Slovenia; 4 Faculty of Medicine, University of Maribor, Taborska ulica 8, 2000 Maribor, Slovenia; 5 Faculty of Health Sciences, University of Maribor, Žitna ulica 15, 2000 Maribor, Slovenia

**Keywords:** difficult intravenous access, intraosseous access, medical education

## Abstract

*Background and Objectives*: Insertion of an intraosseous access device enables intravascular access for critically ill patients in a prehospital and emergency department setting even when intravenous access is not possible. The aim of our study was to assess the attitudes of prehospital and emergency department nursing staff towards the utilization of intraosseous access devices. *Materials and Methods*: We performed quantitative research using a closed-ended structured questionnaire distributed to prehospital unit and associated emergency department nursing staff serving a population of around 200,000 inhabitants. *Results*: We distributed 140 questionnaires, and 106 were returned and completed. Of these, 69 (65.1%) respondents needed more than three attempts to achieve peripheral intravenous access at least once in the last year and 29 (27.4%) required central venous access because of impossible intravenous access. In the last five years, 8 (7.5%) respondents used endotracheal route for administration of medications. Despite this, only 48 (45.3%) of respondents have ever used the intraosseous route. Also, 79 (74.5%) respondents received at least some training in obtaining IO access; however, 46 (43.4%) answered that education regarding intraosseous access is not sufficient, and 92 (86.8%) answered that they wanted additional training regarding intraosseous access. *Conclusions*: Prehospital and emergency department nursing staff are aware of the importance of intraosseous access and understand the need for additional education and certification in this field.

## 1. Introduction

Obtaining intravascular access is paramount in the treatment of critically ill and injured patients [1], and delays in achieving intravascular access are reflected in delays in administration of potentially life-saving therapy [2]. In most cases, intravenous (IV) access is the preferred route of achieving intravascular access [3,4]. However, obtaining IV access is difficult, time-consuming, or impossible in some patients. European Resuscitation Council guidelines recommend intraosseous (IO) access in patients with difficult IV access [5]. In recent years IO access has emerged as a rapid, safe, and effective means of obtaining intravascular access and an effective alternative to IV access [6]. However, the uptake of IO access by providers of prehospital and emergency department care has been slow [7]. The aim of our paper is to report on the attitudes towards the use of IO access of nursing staff serving in a prehospital unit and an emergency department in an area where IO access has been rarely used.

## 2. Materials and Methods

We chose a quantitative research method and performed a survey of nursing staff working in a prehospital unit and an emergency department serving an area with around 200,000 inhabitants. Prehospital unit was affiliated to the same hospital as the emergency department. We obtained institutional ethics committee approval (No. 02/010/03-027/01/20), and informed consent of respondents was waived because of the voluntary nature of the study. We used a closed-ended structured questionnaire with questions based on a literature review [8,9,10], composed by all authors. The questionnaire was distributed to 140 registered nurses of one prehospital unit and one emergency department. The questionnaires were distributed to registered nurses who were not on long-term leaves (for medical causes, vacation or other). Returned and completed survey questionnaires were analyzed and processed using computer programs Microsoft Excel and IBM SPSS 22.0 (IBM Corp., New York, NY, USA). We used descriptive statistics to present the data, namely frequencies (*n*) and associated percentages (%), minimum (Min), maximum (Max), average values (M), and standard deviations (SD) of responses. To discover statistically significant differences in the distribution of responses between groups, we used the Chi-square test with α = 0.05. If the statistical characteristic (*p*-value) was lower than *p* < 0.05, we concluded that statistically significant differences in the distribution of responses between groups do exist, at a risk level of 5%. Invalid or missing answers were excluded from statistical analyzes and data processing.

## 3. Results

### 3.1. Baseline and Demographic Data

The study sample consisted of 106 respondents from the initial 140 mailed questionnaires (return rate 75.7%). Of these, 75 (70.8%) respondents were male, 36.2 ± 9.7 years old, with 14 ± 10.3 years of work experience; 64 (60.4%) respondents were employed in a prehospital unit, and 42 (39.6%) were employed in a hospital-based emergency department (Table 1).

In the year 2020 (one full calendar year before the questionnaires were sent), 13,904 patients were treated in the prehospital setting (2633 interventions with a doctor on board and 11,271 interventions without) by a team comprising 87 registered nurses and 18 doctors. In the same year, 28,484 patients were treated in the emergency department by a team comprising 88 registered nurses and 37 doctors (10 specialists and 27 residents).

### 3.2. Questions Regarding Previous Operator Experience

Based on the responses, 65.1% of respondents required more than three attempts at establishing IV access at least once over the last year; 50% of respondents answered that they usually required less than 1 min to establish an IV access, 42.5% answered that they usually required less than 2 min, 5.7% answered that they required less than 3 min, and 1.9% answered that they required more than 3 min to establish an IV access (Table 1). 

Further, 27.4% of respondents answered that insertion of a central venous catheter was required to establish an intravenous access. Over the last 5 years, 7.5% of respondents utilized an endotracheal administration of therapy during a resuscitation attempt because IV access was not established (Table 1).

A majority of respondents (80.2%) answered that they rarely faced the need to insert an IO access (Table 1).

### 3.3. Questions Regarding Previous Experience with Intraosseous Access

Regarding utilization of an IO access, 54.7% of respondents answered that they have never used it, and 45.3% answered that they used IO access at least once (13.2% used it once, 12.3% twice, 6.6% three times, and 13.2% more than four times). Also, 47.9% of respondents who had set up an IO access at least once decided to attempt an IO access after three unsuccessful attempts at IV access; 18.8% of respondents who have used IO access at least once made the decision to proceed to IO access after three unsuccessful attempts at securing an IV access, 14.6% after two attempts, and 18.8% of respondents decided to use IO access immediately because of circumstances (Table 2).

Different devices have been used for IO access: EZ-IO device in 52.1% of attempts, BIG device in 43.8% of attempts, NIO device in 27.1% of attempts, and FAST device in 6.3% of attempts. Unsuccessful attempts at insertion of IO devices occurred in 12.5% of attempts: in two patients, the device failed to eject; in one patient, the needle twisted; in one patient, the mandrel could not be withdrawn; in one patient, aspiration of bone marrow was not successful; and in one patient, successful insertion was not possible because of obesity (Table 3).

### 3.4. Questions Regarding Intraosseous Access Training

Results indicated that 74.5% of respondents had received at least some training in obtaining IO access. Training was organized by the employer in 74.7% of respondent cases, by the suppliers of IO devices in 30.4% of respondent cases, during formal education in 5.1% of respondent cases, at an advanced life support course in 16.5% of respondent cases and in a specialized IO course in 12.7% of respondent cases (Table 4).

When asked regarding the most optimal location for IO access, 60.6% of respondents answered proximal tibia, 16.3% answered distal tibia, 4.8% answered distal femur, 2.9% answered head of humerus, 4.8% answered sternum, and 13.5% answered “I don’t know” (Table 5).

Slightly less than half (43.4%) of respondents answered that education regarding IO access in Slovenia is not sufficient, slightly more than a quarter (27.4%) answered that education is sufficient, and the rest (29.2%) answered with “I don’t know”. More than half of respondents (60.4%) were not satisfied with their knowledge regarding IO access, and a great majority (86.8%) of respondents wanted additional training and certification of competence regarding IO access (Table 6).

Respondents who reported that they have already inserted an IO access were statistically significantly more likely to have greater satisfaction regarding their knowledge regarding insertion of IO access (76.2% vs. 23.8%, *p* < 0.001) (Table 7).

It was also statistically significantly more likely for respondents who answered that it made sense to insert IO access to have answered that training regarding IO access is important (78.3% vs. 21.7%, *p* = 0.024) (Table 8).

Respondents who have inserted more than three IO needles were not statistically significantly more likely to have required more than three attempts at IV access (*p* = 0.114) (Table 9).

We were also interested in whether there were differences in the proportion of respondents who were trained to set up an IO access according to the age (younger and older) of the respondents. For this purpose, we combined respondents into two age groups, namely younger (up to 35 years; 46.2%) and older (35 years or more; 53.8%). We did not observe any differences between younger and older respondents (75.5% vs. 73.7%, *p* = 0.830) (Table 10).

## 4. Discussion

In our sample population of prehospital and emergency department personnel, 45.3% of respondents answered that they used an IO device at least once during their professional careers. Respondents who have used an IO access previously were more likely to have greater satisfaction regarding their knowledge regarding IO access. Respondents who utilized IO more frequently were not more likely to have required a greater number of attempts at securing IV access. In all, 60.4% of respondents were not satisfied with their knowledge regarding IO access, and a great majority (86.8%) of respondents wanted additional training and certification of competence regarding IO access.

Use of IO access is characterized by high first-attempt success rate even in patients with cardiac arrest, without the requirement for discontinuation of cardiopulmonary resuscitation during insertion [11,12,13]. In patients where difficult IV access can be anticipated (e.g., children, extreme obesity, edema, hypotension, burns, shock, chemotherapy, dehydration, intravenous drug users), IO access can be used as a first choice for obtaining intravascular access [14,15]. IO access can be used for administration of fluids, medications, and blood products [12,13], and if pressurized infusion systems are used, flow rates similar to those achieved in central venous lines can be expected [1,16]. The usual indication for IO access is failed IV access in two or three attempts within two minutes [11]. Complications associated with IO access occur in around 10% of patients with IO access, and successful insertion of IO needles is reported in around 70–100% of patients [17]. In a setting of out-of-hospital cardiac arrest, tibial IO access was found to have higher first-attempt success rate for vascular access (91% vs. 43%) and shorter time to vascular access (4.6 min vs. 5.8 min) compared to peripheral IV access [18].

Reported incidence of difficult IV access varies widely, between 6% and 88% [19]. Additionally, in case of difficult IV access, time for administration of therapy, contrast medium for radiological diagnostic procedures, and laboratory results all increase by 30–60 min [20]. Even in a setting of a prospective, multicenter, interventional trial with the aim to administer adrenaline during out-of-hospital cardiopulmonary resuscitation as per guidelines, time to administration of adrenaline was around 15 min [21].

Even though insertion success rates are high and complications are rare, IO access is routinely used only rarely [4,13]. In a series including 322 patients with critical illness or injury and failed IV access, IO access was used in only 14 patients. Similarly, Smereka et al. reported that only 7.1% of nurses have performed an IO access, and only 10.9% have taken part in any training regarding IO access [7]. Ingrained habits, lack of knowledge and experience, lack of equipment and skills, and ignorance of the benefits have been cited as reasons for low uptake [8,13,22]. In our study, two-thirds of respondents required more than three attempts at securing IV access at least once in the last year, and one-third reported that central venous access was required to secure intravascular access. Also, one tenth of respondents have used endotracheal administration of life-saving medications in the last five years, even though the use of endotracheal access has not been advocated since 2015 [23]. Our results regarding the timing of use of an IO are comparable to other studies. Bloch et al. [24] have reported that 42% of respondents chose IO access only after the fourth attempt at securing IV access.

Multiple intraosseous devices are available. Our respondents reported that the most commonly used devices were the Bone Injection Gun–BIG (PerSys Medical, Houston, TX, USA) and EZ-IO (Teleflex, Morrisville, NC, USA) devices, while Next-Generation IO-NIO (PerSys Medical, Houston, TX, USA) and FAST1 (Teleflex, Morrisville, NC, USA) devices have been used rarely. BIG, NIO, and FAST1 devices are disposable spring-loaded devices comprising one (in case of the BIG and NIO devices) or multiple needles (in case of the FAST1 device) that penetrate the bone after triggering the device [3,4,13]. The EZ-IO device consists of a reusable battery-powered drill and a disposable needle [4]. The FAST1 device is registered for use in the sternum of adult patients, while other devices are most commonly used in the proximal tibia and the head of the humerus and are offered in different sizes for adult and pediatric patients [3,13]. Differences between devices outline the need for device-specific training in addition to general intraosseous access courses.

The proportion of respondents who have received some training in securing IO access is also comparable to other authors. Wolfson et al. [25] have reported that 72% of emergency medicine programs recommend or conduct IO access training. Despite a relatively high percentage of respondents having taken part in at least some IO access training, a great majority of our respondents wanted additional training.

Different approaches to training in securing IO access result in different levels of proficiency; successful insertion rate is 37% if education does not include hands-on training, 65% if traditional needles are used during training, and 97% if novel semi-automatic systems are used during training [26,27]. High-fidelity simulation has been successful in increasing proficiency in other fields in medicine as well. In our case, we have also shown that respondents who are satisfied with their knowledge are more likely to utilize IO access and at the same time are not more likely to require more attempts at securing IV access.

We performed a structured questionnaire survey with all the relevant limitations. We included personnel from two associated units covering a relatively small population and our results can only be generalized with caution. However, there are only a few studies covering this field. Difficult or impossible IV access remains an important problem and our respondents seem to point to more education to alleviate it.

## 5. Conclusions

We have shown that prehospital and emergency departments personnel understand the importance of IO access and express the willingness to attend additional training despite a relatively high percentage of respondents having attended at least some training in securing IO access. This probably points to the need for a more structured and simulation-based approach to education regarding IO access.

## Figures and Tables

**Table 1:** Questions regarding previous operator experience.

Question	Answer	*n*	%
During the past year have you ever required 4 or more attempts at inserting peripreral intravenous access?	Yes	69	65.1
	No	37	34.9
	All	106	100
How long do you estimate it takes you to insert one intravenous access?	<1 min	53	50
	1–2 min	45	42.5
	2–3 min	6	5.7
	4 or more minutes	2	1.9
	All	106	100
During the past year have you ever required a central venous catheter for intravascular administration of medications?	Yes	29	27.4
	No	77	72.6
	All	106	100
During the past 5 years have you required endotracheal administration of medications?	Yes	8	7.5
	No	98	92.5
	All	106	100
How often do you need intraosseous access to achieve intravascular access?	Never	10	9.4
	Rarely (1–5 times per year)	85	80.2
	Sometimes (6–10 times per year)	7	6.6
	Often (11 or more times per year)	4	3.8
	Skupaj	106	100

Legend: *n* = number of answers, % = percentage.

**Table 2:** Questions regarding previous experience with intraosseous access.

Question	Answer	*n*	%
How many times have you used intraosseous access?	Never	58	54.7
	Once	14	13.2
	Twice	13	12.3
	Three times	7	6.6
	Four times or more	14	13.2
	All	106	100
After how many failed attempts to insert an intravenous access have you attempted to insert an intraosseous access?	Immediatelly or after one attempt	9	18.8
	After two attempts	7	14.6
	After three attempts	9	18.8
	After four or more attempts	23	47.9
	All	48	100
If you have ever attempted an intraosseous access, which device did you use?	Bone Injection Gun–BIG	21	43.8
	EZ–IO	25	52.1
	NIO	13	27.1
	FAST1	3	6.3

Legend: *n* = number of answers, % = percentage.

**Table 3:** Questions regarding problems with intraosseous access.

Question	Answer	*n*	%
If you have ever attempted an intraosseous access, have you experienced any problems?	Yes	6	12.5
	No	42	87.5
	All	48	100

Legend: *n* = number of answers, % = percentage.

**Table 4:** Questions regarding intraosseous access training.

Question	Answer	*n*	%
Have you participated in any training regarding intraosseous access?	Yes	79	74.5
	No	27	25.5
	All	106	100
If you have participated in any training, in which setting did it take place ?	During formal education	4	5.1
	Informally during work	29	36.7
	Formal education organized by the employer	30	38
	Education by the dealer of the equipment	24	30.4
	At the advanced life support course	13	16.5
	Other	10	12.7

Legend: *n* = number of answers, % = percentage.

**Table 5:** Questions regarding location of intraosseous access.

Question	Answer	*n*	%
If you have ever attempted an intraosseous access, which anatomical location did you use?	Proximal tibia	63	60.6
	Distal femur	5	4.8
	Proximal humerus	3	2.9
	Distal tibia	17	16.3
	Sternum	5	4.8
	Other	1	1
	I don’t know	14	13.5

Legend: *n* = number of answers, % = percentage.

**Table 6:** Questions regarding education in intraosseous training.

Question	Answer	*n*	%
Do you think that using intraosseous access is reasonable in your work setting?	Yes	92	86.8
	No	6	5.7
	I don’t know	8	7.5
	All	106	100
Do you think that education regarding intraosseous access is accessible enough?	Yes	29	27.4
	No	46	43.4
	I don’t know	31	29.2
	All	106	100
Are you satisfied with your knowledge regarding intraosseous access?	Yes	42	39.6
	No	64	60.4
	All	106	100
Would you like to undergo a certification of competence process regarding intraosseous access?	Yes	92	86.8
	No	8	7.5
	I don’t know	6	5.7
	All	106	100

Legend: *n* = number of answers, % = percentage.

**Table 7:** Satisfaction with knowledge regarding intraosseous access vs. number of insertions of intraosseous access.

Variable	Answer	*n* %	Are You Satisfied with Your Knowledge Regarding Intraosseous Access?		Chi-Square Test
			Yes	No	
How many times have you used intraosseous access?	Once or more	*n*	32	16	*p* < 0.001
		%	76.2	25	
	Never	*n*	10	48	
		%	23.8	75	
All		*n*	42	64	
		%	100	100	

Legend: *n* = number of answers, % = percentage.

**Table 8:** Using intraosseous access makes sense vs. participation in any training.

Variable	Answer	*n* %	Do You Think That Using Intraosseous Access Is Reasonable in Your Work Setting?		Chi-Square Test
			Yes	No/I Don’t know	
Have you participated in any training regarding intraosseous access?	Yes	*n*	72	7	*p* = 0.024
		%	78.3	50	
	No	*n*	20	7	
		%	21.7	50	
All		*n*	92	14	
		%	100	100	

Legend: *n* = number of answers, % = percentage.

**Table 9:** Questions regarding problems with intraosseous access.

Variable	Answer	*n* %	During the Past Year Have You Ever Required 4 or More Attempts at Inserting Peripreral Intravenous Access?		Chi-Square Test
			Yes	No	
How many times have you used intraosseous access?	Never	*n*	35	23	*p* = 0.114
		%	50.7	62.2	
	Once or twice	*n*	22	5	
		%	31,9	13.5	
	Three or more times	*n*	12	9	
		%	17.4	24.3	
All		*n*	69	37	
		%	100	100	

Legend: *n* = number of answers, % = percentage.

**Table 10:** Questions regarding problems with intraosseous access.

Variable	Answer	*n* %	Age		Chi-Square Test
			Under 35 Years	35 Years and Older	
Have you participated in any training regarding intraosseous access?	Yes	*n*	37	42	*p* = 0.830
		%	75.5	73.7	
	No	*n*	12	15	
		%	24.5	26.3	
All		*n*	49	57	
		%	100	100	

Legend: *n* = number of answers, % = percentage.

## Data Availability

Study data is available via the corresponding author.
